# MIGRATION AND COGNITIVE HEALTH DISPARITIES: THE ARAB AMERICAN AND REFUGEE CASE

**DOI:** 10.1093/geroni/igae098.1193

**Published:** 2024-12-31

**Authors:** Tala Al-Rousan, Alison Moore, Alissa Bernstein Sideman, Kristine Ajrouch, Lily Kamalyan

**Affiliations:** University of California San Diego, La Jolla, California, United States; University of California San Diego School of Medicine, San Diego, California, United States; University of California San Francisco, San Francisco, California, United States; University of Michigan, Ann Arbor, Michigan, United States; San Diego State University/University of California San Diego Joint Doctoral Program in Clinical Psychology, San Diego, California, United States

## Abstract

This study investigates whether the year of arrival to the United States (U.S.) and birthplace relate to postmigration cognitive difficulties among foreign- and U.S.-born Arab Americans in later life. We analyzed 19 years (2000-2019) of data from the American Community Survey Public Use Microdata Samples (weighted N = 393,501; ages ≥ 50 years). The cognitive difficulty was based on self-reported data and weighted means, percentages, adjusted prevalence estimates, and adjusted odds ratios were calculated. Controlling only for demographics, foreign-born Arabs reported higher odds of cognitive difficulty compared to U.S.-born Arabs across all arrival cohorts (p <.001). After accounting for economic and integration factors, those who arrived between 1991 and 2000 had higher odds (odds ratio [OR] = 1.06, 95% confidence interval [CI] =1.00, 1.19, p <.01), while those who arrived after 2001 had lower odds (OR = 0.87, 95% CI = 0.78, 0.97, p <.001) of cognitive difficulty. Lacking English proficiency (OR = 1.90, 95% CI = 1.82, 1.98, p <.001) was related to higher odds, whereas not being a U.S. citizen was significantly associated with lower odds (OR = 0.89, 95% CI = 0.52, 0.94, p <.001) of cognitive difficulty. Yet, results varied by birthplace. Migrants born in Iraq consistently reported the highest odds of cognitive difficulty across all arrival cohorts. Migration history and birthplace are essential factors explaining cognitive disparities among the diverse group of Arab migrants and Arab Americans. Reasons for migration such as displacement due to war may explain these disparities.

